# Climate change anxiety, fear, and intention to act

**DOI:** 10.3389/fpsyg.2024.1341921

**Published:** 2024-02-29

**Authors:** Alessandro von Gal, Greta Fabiani, Laura Piccardi

**Affiliations:** ^1^Spatial Cognition Lab, Department of Psychology, Sapienza University of Rome, Rome, Italy; ^2^San Raffaele Cassino Hospital, Cassino, Italy

**Keywords:** climate change anxiety, eco-anxiety, eco-emotions, framing, sustainability, Bayesian analysis

## Abstract

Climate change anxiety (CCA) is an emerging yet not clearly defined construct. Here, we examine the relationship between CCA and climate change-related fear in response to messages differently framing uncertainty and anticipation concerning climate change, exploring how the former differs from general anxiety measures. To this purpose, young and healthy volunteers were assigned to three different framing conditions. Their emotional responses as well as eco-emotions and beliefs about climate change were collected. By employing a Bayesian approach, we found that framing the consequences of climate change effectively induces heightened fear and that CCA strongly predicted fear levels, while general anxiety measures did not. Overall, these results reflect CCA’s unique and specific nature in influencing climate change-related fear. Interestingly, we found fear to predict intention scores only following the framings that did not effectively induce action intentions, consistent with prior findings on fear without efficacy framing. Instead, reading about the negative consequences motivated action the most. Following this framing, we found that eco-anger, instead of fear, consistently predicted intentions to engage in climate action. These results emphasize the complex interplay between CCA, eco-emotions, efficacy, and behavioral engagement.

## 1 Introduction

Climate change is one of the toughest challenges that mankind has ever faced. The Intergovernmental Panel on Climate Change (IPCC, 2023) sixth assessment report unequivocally confirms the significant anthropogenic impact on atmospheric, oceanic, and terrestrial warming, resulting in widespread and rapid changes across the atmosphere, oceans, cryosphere, and biosphere. This human-induced warming has occurred at an unprecedented rate within the past 2000 years, primarily due to an unparalleled increase in mixed greenhouse gas emissions. In assessing the possible future scenarios, the report underlines the urgent necessity of reducing greenhouse gas emissions to remain below the critical threshold of a 1.5° increase (compared to pre-industrial levels) to irreversible consequences.

Despite the increasing scientific efforts in describing and predicting the consequences of climate change, people’s beliefs about them can vary widely, and the relationship between these beliefs and supporting or engaging in adaptive behaviors is not completely clear (van Valkengoed and Steg, 2019; van Valkengoed et al., 2021). For example, previous research described different types of climate skepticism on a continuum, from skepticism to denial (Rahmstorf, 2004; Poortinga et al., 2011; Capstick and Pidgeon, 2014; Haltinner and Sarathchandra, 2021; de Graaf et al., 2023), and found several social factors like age, gender, ethnicity and political ideology as predictors (Upham et al., 2009; McCright and Dunlap, 2011a,b). While it is straightforward to imagine that the higher the skepticism, the lower the engagement in adaptive actions will be, stronger risk perceptions do increase intentions but not always actual behaviors (van Valkengoed et al., 2023).

To better understand the gap between intentions and behaviors, previous research has concentrated on people’s responses to the future consequences of climate change, which mainly involve uncertainty, unpredictability, and uncontrollability. These feelings are closely associated with Generalized Anxiety Disorder (GAD) symptoms (Pihkala, 2020). Indeed, over the past decade, terms like Eco-anxiety or Climate Change anxiety (CCA) have been proposed to describe anxiety-related symptoms and manifestations in response to climate change as an environmental stressor (Clayton, 2020). Several definitions have been proposed, with compelling reviews (e.g., Clayton, 2020; Pihkala, 2020; Kurth and Pihkala, 2022). Many of these definitions typically refer to the proposals of Albrecht (2011, 2012), who frames eco-anxiety as a generalized, wide-scale reaction to the state of the planetary ecosystems, categorizing it among what he refers to as “psychoterratic” syndromes (Albrecht, 2011). Like general anxiety, CCA is closely linked to feelings of fear and worry but also involves hopelessness and anger, with close connections with frustration, despair, guilt, shame, and grief (Clayton, 2020; Clayton and Karazsia, 2020; Pihkala, 2020; Verplanken et al., 2020). There is still no precise classification of what is meant by “anxiety” in this context (Pihkala, 2020); Coffey et al. (2021) described more than ten distinct operationalizations of this phenomenon, suggesting an evident lack of consensus (Heeren et al., 2023). Regardless, many studies demonstrate the relationship between GAD-related symptoms and CCA (Materia, 2016; Innocenti et al., 2021; Whitmarsh et al., 2022; Asgarizadeh et al., 2023; Schwartz et al., 2023). Notably, anxiety is a fundamental process serving an adaptive function in humans and other animals. It involves negative emotionality characterized by physical symptoms and future-oriented apprehension. As such, it serves as an adaptive response as it leads to preparation for future events and eventual threats (Barlow, 2004). However, such responses may become maladaptive, leading to emotional dysregulation and maintenance of a chronic state of worry and rumination, as in the case of GAD (Barlow, 2004; Barlow et al., 2016). This holds for CCA as well, which should be considered on a continuum (Clayton et al., 2023) with symptoms ranging from mild to severe, potentially resulting in a clinically definable climate anxiety disorder (Pihkala, 2019). In the context of CCA, it is of great importance, as several authors do, to distinguish between adaptive and maladaptive forms to avoid pathologizing the emotional response to climate change (Reser et al., 2012; Clayton, 2020). Indeed, this is particularly relevant when talking about CCA, where the primary stressor and cause of distress is characterized by a tangible and approaching threat that should not be avoided, for which subclinical anxiety is an appropriate response and a potential resource (Pihkala, 2019; Clayton and Karazsia, 2020; Clayton et al., 2023). As such, emotion-focused coping strategies that rely on reframing or de-emphasizing the risk’s threat are unfit to deal with CCA given that the climate-related issues will remain and likely worsen (Clayton, 2020). Thus, this type of coping mechanism is ineffective for the individual and, most importantly, for the aim of dealing with environmental issues, potentially leading to psychological distancing and discouraging individual and collective action.

Accordingly, several studies demonstrated the potentiality of CCA in motivating action (Heeren et al., 2023). Indeed, through a mediation model, Innocenti et al. (2023) demonstrated that the CCA cognitive impairment subdimension simultaneously has an opposite effect on pro-environmental behaviors (PEBs), directly encouraging PEBs but also negatively influencing PEBs through self-efficacy levels, possibly leading to eco-paralysis.

To operationalize and recognize CCA, Clayton and Karazsia (2020) developed the Climate Change Anxiety Scale (CCAS) and found evidence for two domains, a functional and a cognitive one, closely correlated but showing different correlation patterns with other domains. Moreover, they found that negative emotional responses can be distinguished from a clinically defined “anxiety” response since the former was associated with behavioral engagement, differently from CCA measures, concluding that more research is needed to examine the predictors leading to adaptive emotional responses and positive behavioral responses.

Recently, Asgarizadeh et al. (2023) proposed a model of the predictors of CCA using structure equation modeling and found GAD traits to be the most critical ones, followed by prior experience with climate change impacts and climate change knowledge. Among the factors, they highlight the importance of media exposure in mediating the relationship between prior exposure and CCA.

In an attempt to motivate environmental action, ecological information campaigns usually aim at promoting individual behavioral change by concentrating on the disclosure of the negative consequences of climate change. This approach enhances fear levels, consequently motivating pro-environmental action. However, this is true only when strong fear appeals are accompanied by high-efficacy messages, potentially producing the greatest behavioral change. On the other hand, having low-efficacy messages will likely produce the greatest levels of defensive responses instead (Witte and Allen, 2000). Inducing high levels of fear may lead to denial and apathy, resulting in more psychological distancing from the issue (Kollmuss and Agyeman, 2002). These emotional components play a key role in the onset of climate change helplessness – the belief that climate change is beyond personal control, resulting in behavioral inhibition related to pro-environmental actions (Salomon et al., 2017). Conversely, adding indications about what to do, individually and collectively, to mitigate the environmental impact increases efficacy beliefs. This, in turn, improves intentions to conserve the environment and PEBs (van Zomeren et al., 2010; Chen, 2016; Jugert et al., 2016; Salomon et al., 2017; Fritsche and Masson, 2021).

As for CCA, different studies argue for the positive effect of fear of the negative outcomes in motivating action and behavioral change, mainly when framed with efficacy messages (van Zomeren et al., 2010; Tannenbaum et al., 2015; Chen, 2016). As mentioned above, CCA is empirically distinguished from fear, although fear and anxiety often co-occur (Clayton, 2020).

The present study investigates how CCA contributes to the formation of CC-related fear in response to messages differently framing uncertainty and anticipation concerning climate change. Namely, one only discloses the causes of climate change, and another explains the causes and the future consequences. First, we hypothesized the Consequences condition to induce the highest fear levels, replicating van Zomeren et al. (2010) results. After confirming this, our main hypothesis was that participants’ dispositional CCA would predict fear levels induced by the framings, while general measures of anxiety did not. Particularly, we assessed the differential effect of the two subscales on fear formation and hypothesized that if the previous accounts that underlined the importance of the cognitive-emotional impairment subdimension hold true (Heeren et al., 2023; Innocenti et al., 2023), this would have the highest predictive power on fear formation. Specifically, we also hypothesized a susceptibility effect for which participants with the highest CCA scores would be more affected by the framings that effectively induced eco-fear. Lastly, we observed how increased eco-fear induced by the framings would translate to increased intentions to act pro-environmentally. Specifically, we assessed participants’ perceptions about climate change following the experimental manipulation, and investigated how these, together with fear and CCA would predict people’s intentions to engage in pro-environmental actions. In absence of an efficacy manipulation and based on previous accounts on eco-paralysis and climate-change helplessness, we expected to find moderate levels of fear to be most effective in motivating action, supposedly following the Causes only condition. Conversely, we did not have definitive hypotheses about the role of CCA, given the lack of consensus regarding its relationship on both intentions and actual behaviors on one side, and the dual nature of adaptive and maladaptive anxiety in motivating action on the other.

## 2 Materials and methods

### 2.1 Participants and design

The experiment was conducted online using the Qualtrics platform (Qualtrics, Provo, UT). 265 participants volunteered to participate in the study and received no compensation. All of them were native Italian speakers. University students participated in a course-related activity, while others were recruited through word of mouth. Participants who previously suffered from traumatic brain injuries, epilepsy, or other brain-damaging events; participants who suffered from psychiatric disorders in the last 5 years; who were currently on psychopharmacological treatment or abusing substances were not considered for the analysis, leaving us with 177 participants. During the online survey, reading times corresponding to the experimental texts were collected, and implausible reading times were excluded. For each experimental text, we calculated a threshold of minimum milliseconds required to silently read the whole message based on the number of syllables present in the text. Specifically, we chose to set the fastest acceptable syllables per second rate (syll/s) to 13.03, based on the findings of Ciuffo et al. (2017) on Italian university students silent-reading nonfiction texts, and calculated the minimum plausible reading time for each presented text. After discarding the implausible reading times, we excluded outliers exceeding two standard deviations, for each text message, leaving us with a final sample of 122 participants (50 in the Baseline condition, 39 in the Causes condition and 33 in the Consequences condition). Samples’ descriptives are described in Table 1.

**Table 1:** Descriptive statistics.

Age in years	
Mean (SD)	23.2 (4.7)
Median	22
Range	18–39
Gender	
Male	43 (35.25%)
Female	78 (63.93%)
Preferred not to answer	1 (0.82%)
Years of education	
Mean (SD)	14.2 (2.3)
Median	13
Occupation	
Unemployed	3 (4.1%)
Student	12 (9.8%)
University student	72 (59%)
Employee	24 (19.7%)
Freelance	5 (4.1%)
Other	4 (3.3%)
Status	
Low	20 (16.4%)
Medium	94 (77.1%)
High	6 (4.9%)
Missing	2 (1.6%)
Income	
No income	68 (55.7%)
Less than 1,000 euros per month	27 (22.1%)
More than 1,000 euros per month	15 (12.3%)
More than 1,500 euros per month	6 (4.9%)
More than 2000 euros per month	5 (4.1%)
More than 2,500 euros per month	1 (0.8%)
Total = 122	

After reading and accepting the informed consent form, all participants were asked to fill in their sociodemographic information. Then, participants were asked to fill in a series of questionnaires presented in a randomized order, specifically the State and Trait anxiety scales and the Depression scale from the Cognitive Behavioral Assessment (CBA) scale, the Social Desirability scale, the Climate Change Anxiety scale, and the Positive Affect Negative Affect Schedule (PANAS). After completing these questionnaires, the experimental manipulation consisted of a between-subject design in which participants were randomly assigned to one of three conditions: a neutral Baseline condition in which they read a text not related to climate change, a Causes condition in which they read about the causes of climate change and a Consequences condition in which they read about the future consequences of climate change. Reading times were collected for each presented message in all conditions. Right after the framing, all participants were asked to report how they felt. Namely, we asked them to report the positive or negative valence of the emotion they felt related to the message and how much this activated them. Additionally, they answered the degree to which they felt both anger and fear concerning climate change, as well as how much they were motivated to engage in pro-environmental actions. Lastly, all participants answered the PANAS again to measure the effect of the manipulation on positive and negative affect, followed by a final questionnaire about their beliefs about climate change to assess how these were affected by the framing.

The research was approved by the Department of Psychology, Sapienza University of Rome (Prot. n. 001295), following the Declaration of Helsinki, and the Committee itself monitored the execution and results. Each participant signed the written consent form after the procedures were fully explained.

### 2.2 Materials

#### 2.2.1 Questionnaires

Participants were presented with the computerized versions of the following questionnaires:

Sociodemographic information, including information about the level of education, age, gender, socioeconomic status, etc., and a brief anamnestic questionnaire to exclude participants suffering from self-reported neurological or psychiatric diseases or with use and abuse of substances.

Three scales of the Cognitive Behavioral Assessment (CBA) scale battery (Sanavio et al., 1986; Bertolotti et al., 1990), particularly the two State–Trait Anxiety Inventory (STAI) scales (Pedrabissi and Santinello, 1989) measuring both state (SA) and trait anxiety (TA) levels, each containing 20 items, and the Depression Questionnaire (DQ) scale (Bertolotti et al., 2000) containing 24 items measuring dysphoria and depressive symptoms.

The Social Desirability Scale (Manganelli Rattazzi et al., 2000) contains 33 items used to control and account for the possible tendency of participants to give answers that appear suitable in the context of sustainable choices; this could be a confounding variable given the moral value of the topic at hand.

The Climate Change Anxiety Scale (CCAS) (Innocenti et al., 2021) contains 13 items to assess participants’ anxiety levels related to the future consequences of climate change. The items of this scale are divided into a cognitive-emotional impairment subscale (CCAcog) and a functional one (CCAfun). Specifically, the first 8 items of the scale measure CCAcog and assess the impacts on cognition and emotions, as well as rumination, due to climate change. The remaining 5 items assess how the emotions associated with climate change interfere with peoples’ everyday functioning (Clayton and Karazsia, 2020).

The Italian version of the Positive Affect and Negative Affect Schedule (PANAS) (Terracciano et al., 2003) contains 20 items used to assess the intensity of the current affect.

The Climate Change Perception Scale (CCP), developed by van Valkengoed et al. (2021), contains 24 items and was translated into Italian by the authors and back translated by an external expert to ensure accuracy. This scale assesses participants’ understanding of climate change’s causes, current state, and potential consequences. We treated each subdimension separately as they were developed in the original study; these measure the degree of belief that climate change is real (CCP-R), is mainly caused by humans (CCP-H), is due to natural causes (CCP-N), will bring to negative consequences (CCP-NC) or positive consequences (CCP-PC), will affect someone’s proximal areas (CCP-SP), will affect only spatially distant areas (CCP-SD), will happen shortly (CCP-TD).

#### 2.2.2 Stimuli

**Figure 1:**
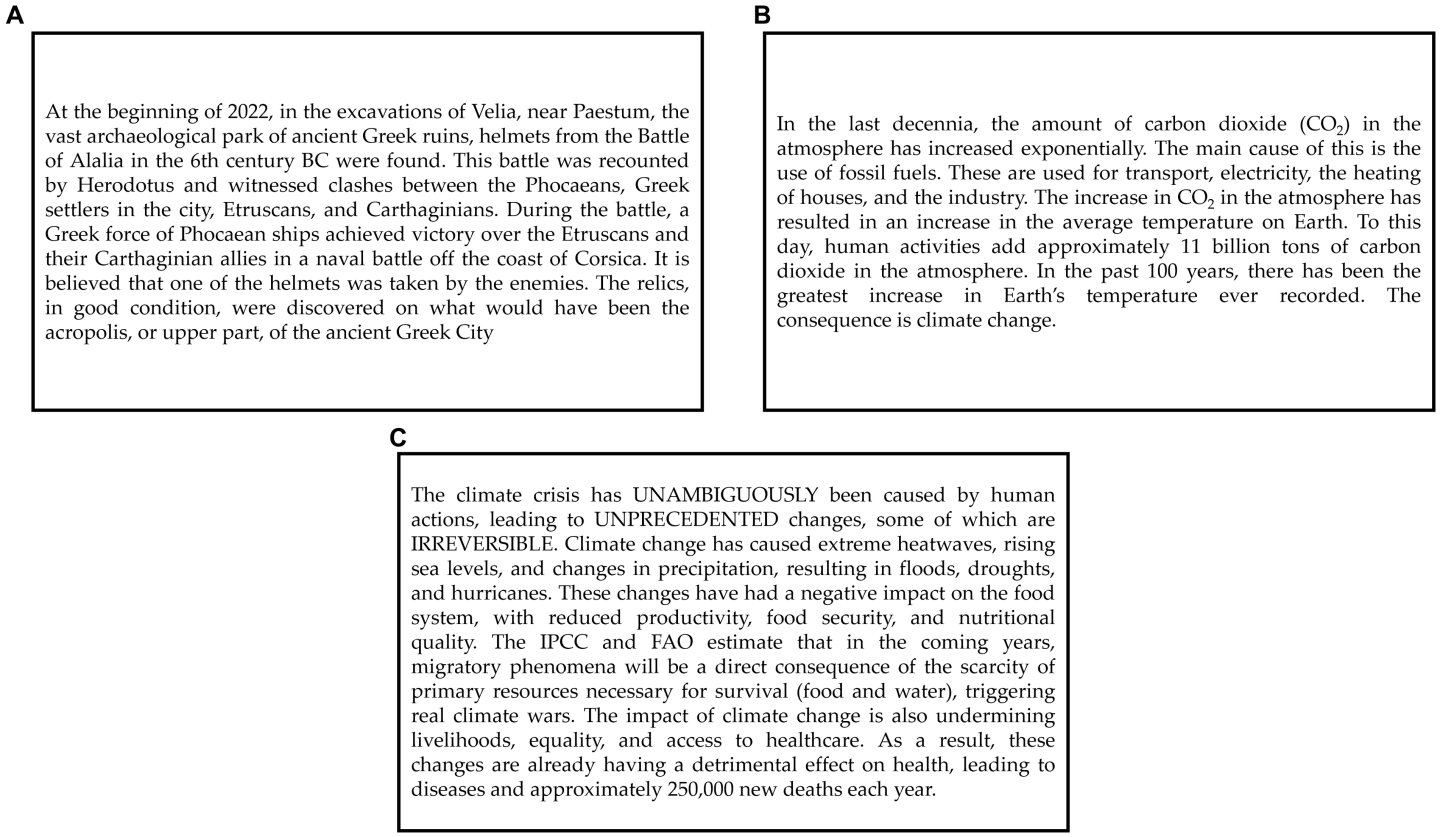
Text messages used in the experiment. **(A)** The neutral message used in the Baseline condition; **(B)** the text describing the causes of climate change used in the Causes condition; **(C)** the text describing the consequences of climate change, presented after the causes text in the Consequences condition.

Participants were randomly assigned to either one of the experimental conditions: in the Baseline condition, participants read neutral archeological information; in the Causes condition, participants read a message talking about the causes of climate change adapted from the one presented in van Zomeren et al. (2010) as the neutral condition; in the Consequences condition, participants first read the same information of the Causes group followed by a message talking about the future negative consequences of climate change. Figure 1 shows the three texts used in the experiment translated from Italian.

The Causes and Consequences messages were accompanied by a sentence stating that the information participants were about to read was taken from the last report of the International Panel on Climate Change (IPCC).

#### 2.2.3 Dependent variables

After reading the three messages, participants responded to 7-point response scale measures (1 = not at all, 7 = very much). Two items measured climate change-related fear (“I am fearful/afraid of the negative future consequences of the climate crisis”), three measured anger related to climate change consequences (“I feel angry/ furious/mad because of the negative future consequences of the climate crisis”) and an additional three items measured intentions to act (“I would like to do something together with others to fight the climate crisis/I would like to sign a petition to promote measures against the climate crisis/I will vote for a political party that fights against the climate crisis”). All these items were taken from van Zomeren et al. (2010). To assess the effect the framings had on emotional valence and emotional arousal, we asked participants to report whether the emotions they felt right after reading the messages were positive or negative, and how much they felt activated by using the classic Self-Assessment Manikin (SAM) (Bradley and Lang, 1994) scored from 1 to 5.

### 2.3 Procedure

All the questionnaires and tasks were presented on the online survey platform Qualtrics (Qualtrics, Provo, UT). The experiment was structured as follows: participants, after accepting and signing informed written consent, were first presented with the sociodemographic and anamnestic questionnaires, then they were presented with the CBA subscales, social desirability, CCAS and PANAS questionnaires. After answering these, they were randomly assigned to one of the three conditions and answered the same Fear, Anger and Intentions items. There was no time limit for the presentation of the different messages; participants could choose when to move on to the next page. After this, all participants answered the 8 subscales of the CCPS and the post-manipulation PANAS.

### 2.4 Statistical analyses

Bayesian hypothesis testing and parameter estimation using JASP, Version 0.17.2.1 (JASP Team, 2024) was used for all the present analyses. All the annotated .jasp files, including the main analyses and manipulation checks, are available at https://osf.io/sydz2/. To test our first main hypothesis, investigating the specific contribution of CCA on Fear in response to the three framings, we conducted a Bayesian ANCOVA comparing Fear scores among the three groups and added CCA, SA and TA scores as covariates, together with three other predictors modeling the interaction between condition and anxiety scores to test for a “sensitivity” effect. Following this analysis, we conducted a similar ANCOVA including the functional and cognitive impairment subdimensions of CCA, and the respective interaction terms, to explore the specific contribution of the two dimensions in eco-fear formation. Furthermore, we tested whether the framings would effectively motivate environmental action by running another ANOVA on intention scores among the three conditions, expecting that moderate fear levels would induce the highest action intentions. Notably, we followed up this analysis by running three separate Bayesian multiple regressions to understand how the different framings influenced individuals’ perceptions of climate change (assessed using the CCP scale) and how these, together with eco-emotions and participant’s dispositional CCA, would predict intentions to act. For all ANOVA and ANCOVA analyses, we assigned equal probabilities to both the null model and the model of interest using a uniform distribution. For the regression analyses, we used a Beta-Binomial model prior (*α* = 1, *β* = 1) to model the parameters’ prior distributions. We employed the default Jeffreys-Zellner-Siow (JZS) prior with an *r* scale of 0.354 due to its reliability in Bayesian analysis (Rouder and Morey, 2012; Ly et al., 2016). Markov chain Monte Carlo (MCMC) was employed as the sampling method, with 1,000 iterations. Robustness checks, in which the same regression was conducted with “wide” and “ultra-wide” priors by changing the *r*-scale to 0.25 and 0.5, can be found in the corresponding .jasp files.

## 3 Results

### 3.1 Controlling for confounding variables and manipulation checks

Before conducting the main analyses, we checked whether other confounding variables may have explained differences between groups. The results of the Bayesian one-way ANOVA revealed that participants in the three groups did not differ in terms of trait (BF_10_ = 0.096) and state anxiety (BF_10_ = 0.192), depression (BF_10_ = 0.281), climate change anxiety (BF_10_ = 0.139) and social desirability (BF_10_ = 0.120).

#### 3.1.1 Effect on mood and affect

To check whether our stimuli induced differences in participants’ mood, we conducted a Time (before/after) x Condition (baseline/causes/consequences) mixed ANOVA model comparing participant’s positive and negative affect scores before and after the presentation of the stimuli. Participants in the three conditions did not differ in neither positive (BF_10_ = 0.695) nor negative (BF_10_ = 0.273) affect scores before being assigned to the respective conditions (i.e., T0).

Regarding the positive affect scores, the analysis of effects resulting from the Time x Condition mixed ANOVA reveals a strong effect of Time (BF_incl_ = 3.272 × 10^+6^) and no effect of both Condition alone (BF_incl_ = 0.858) and Time x Condition interaction (BF_incl_ = 0.323). Conversely, the same analysis conducted on the negative affect scores revealed that the observed data were more likely to occur under the null model (posterior odds of 0.349, BF_M_ = 2.141) however this resulted to be only around 1.08 times more likely than the full model (posterior odds of 0.321, BF_M_ = 1.893). This indicates that results are inconclusive: there is no consistent evidence in favor of one model over the other, possibly due to the considerable variability of NA scores in the three groups. The analysis of effects also reflects this, and shows only limited evidence for the three predictors; with the Time x Condition interaction term (BF_incl_ = 1.893) being the only showing an increase in posterior odds given the data, compared to the Condition (BF_incl_ = 0.635) and Time (BF_incl_ = 0.771) predictors. To better understand this point, we plotted the shift between T0 and T1 scores across all three conditions (available on the OSF repository), and we noticed that the Causes condition showed the highest increase between the two time points. Therefore, we followed up the previous analysis and conducted a one-tailed paired sample *t*-test for each condition to test the alternative hypothesis that NA scores would be higher at T1 compared to T0, with a default Cauchy prior (scale = 0.707). After correcting for multiple comparisons using the Westfall method (Westfall and Wolfinger, 1997), we found the Causes condition to be the only showing a BF favoring the alternative hypothesis for which scores at T0 would be lower than scores at T1 (BF_-0U_ = 15.291; BF_−0_ = 15.291*0.260 = 3.976). Moreover, we wanted to assess how the framings influenced participants’ reported levels of emotional valence and arousal. Namely, we expected the Consequences condition to elicit a decrease in emotional valence and increased emotional arousal compared to the Causes condition and the Baseline condition. Regarding the effect of condition on emotional valence, the one-way Bayesian ANOVA confirmed that the observed emotional valence scores are extremely more likely under the alternative hypothesis predicting a differential effect due to condition, compared to the null model, which represents the null hypothesis of having no differences between conditions (BF_10_ = 2.479 × 10^+9^, 0.011% error). We conducted post-hoc pairwise comparisons to investigate the differences between groups; specifically, the adjusted posterior odds demonstrate extreme evidence for decreased emotional valence in the Consequences condition compared to the Baseline (i.e., odds of 1.413 × 10^+7^ with 1.4 × 10^−13^% error) as well as the Causes condition compared to the Baseline (i.e., odds of 88690.645 with 8.177 × 10^−8^% error). Moreover, the analysis revealed only limited evidence for lowered emotional valence scores in the Consequences condition compared to the Causes (i.e., odds of 1.684 with 0.008% error), indicating that these two conditions induced similar levels of negative emotionality. Regarding emotional arousal scores, the Bayesian ANOVA revealed moderate evidence in favor of the effect of Condition on arousal scores (BF_10_ = 3.544 with 0.028% error). Particularly, the post-hoc comparisons showed that the only consistent difference was found between the Consequences and the Causes condition (adjusted posterior odds of 6.136 with 3.269 × 10^−7^%error).

#### 3.1.2 Effect on climate change-related fear and anger

**Figure 2:**
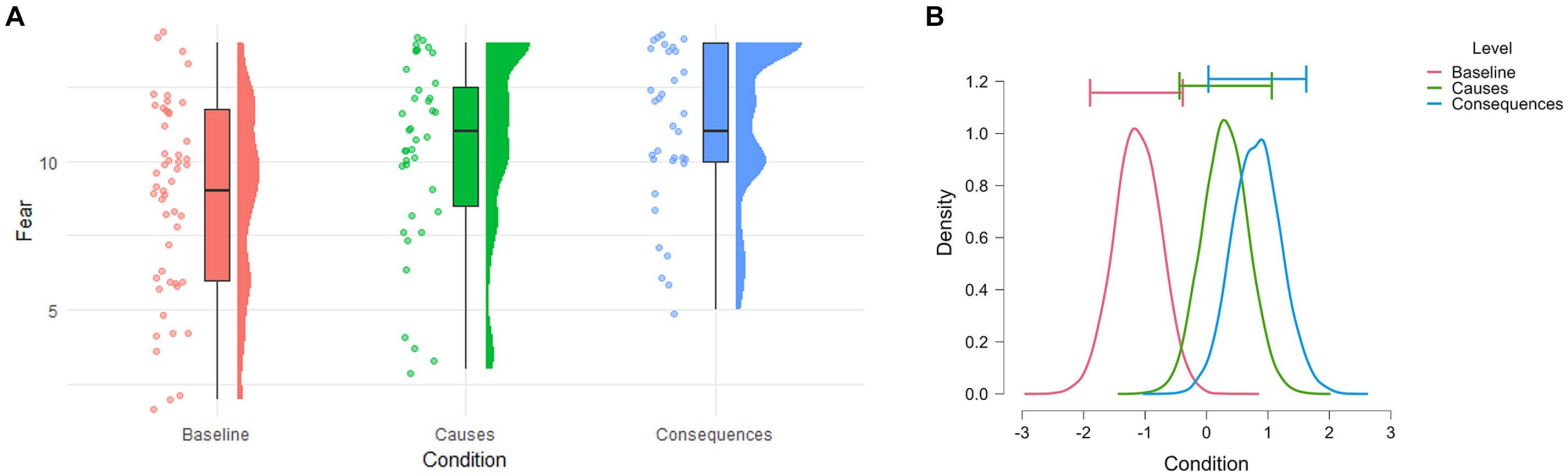
**(A)** Raincloud plot of the observed fear scores in the three Conditions. **(B)** Posterior distributions of the effect of each condition on fear scores. The horizontal error bars represent 95% credible intervals.

Before investigating the relationship between eco-fear and CCA we had to confirm whether our manipulation was effective and specifically induced fear, thus, we used the same eco-anger items that were used in van Zomeren et al. (2010). However, the Bayesian ANOVA comparing the scores on anger in the three groups revealed that data were slightly more probable to occur under the alternative hypothesis of having differences between the groups (BF_10_ = 1.858 with 0.023% error). Particularly, after running pairwise post-hoc comparisons, the adjusted posterior odds demonstrate evidence in favor of a difference between the Consequences condition and the Baseline (i.e., odds of 3.487 with 6.848×10^−7^% error), evidence that the scores did not differ between the Causes and Consequences conditions (i.e., odds of 1/0.402 ≃ 2.49), and moderate evidence that the levels of anger between the Causes and Baseline is also the same (i.e., odds of 1/0.241 ≃ 4.15). Overall, the anecdotal model comparison BF and the post-hoc tests indicate that the conditions did not consistently induce differences in anger levels. Conversely, we assessed the effectiveness in inducing eco-fear with another one-way Bayesian ANOVA, which revealed substantial evidence in favor of the alternative hypothesis (BF_10_ = 10.250 with 0.031% error), indicating a difference in fear scores among the three groups (Figure 2). The post-hoc comparisons revealed moderate evidence indicating heightened fear scores in the Consequences condition compared to Baseline (odds of 11.792 with 1.563 × 10^−7^% error) and very small evidence for heightened fear scores in the Causes-Baseline comparison (odds of 1.423 with 0.009% error). Notably, the analysis revealed moderate evidence that the two groups of interest (Causes vs. Consequences) induced the same levels of climate change-related fear (odds of 1/0.198 ≃ 5.05 with 0.013% error).

### 3.2 Climate change anxiety and climate change-related fear

**Figure 3:**
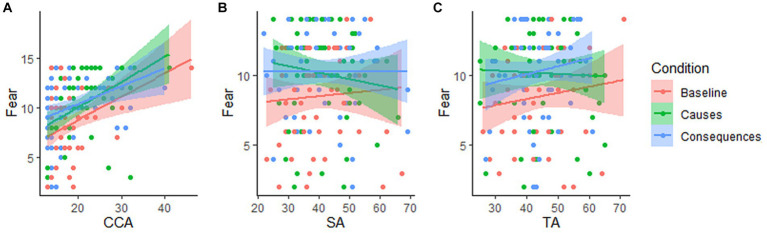
Scatter plots showing the relationship between scores on **(A)** climate change anxiety, **(B)** state anxiety, **(C)** trait anxiety and fear scores in all three conditions. A clear linear relationship emerges only for CCA scores across all conditions.

The main focus of the study was to investigate how higher levels of CCA would influence susceptibility to the two types of climate change messages. Here, we expected higher levels of climate change anxiety to interact with seeing the climate change-related messages in inducing higher levels of fear, and this interaction would be specific for CCA but not for general anxiety levels. Thus, following up on the previous analysis, we conducted a Bayesian ANCOVA with climate change-related fear as the dependent variable and CCA, SA, and TA scores as covariates, to test our hypothesis of a specific involvement of CCA in fear formation, independently of participants’ general anxiety levels. Specifically, we also modeled three more predictors representing the interaction between the condition and each anxiety scale to assess the presence of a “sensitivity” effect. The best model predicting fear scores was the one combining Condition and CCA for which we found extremely strong evidence compared to the null hypothesis (posterior odds of 0.405, BF_10_ = 1560.73 with 0.941 error %). Table 2 shows the analysis of effects across all models to estimate predictors’ inclusion probabilities given the data. Indeed, the most important predictor was CCA (posterior odds of 0.995, BF_incl_ = 117.037) while evidence was against both SA and TA as predictors of eco-fear scores. These latter results confirmed our hypothesis about a relationship between eco-fear and CCA, independently from general anxiety measures: Figure 3 depicts how fear varies linearly as a function of CCA for each condition, compared to SA and TA where there is no linear relationship.

**Table 2:** Analysis of effects summarizes the effect of the different predictors (condition, climate change anxiety, state and trait anxiety) on fear levels.

Effects	P(incl)	P(excl)	P(incl|data)	P(excl|data)	BF_incl_
Condition	0.771	0.229	0.880	0.120	2.167
CCA	0.629	0.371	0.995	0.005	117.037
SA	0.629	0.371	0.247	0.753	0.194
TA	0.629	0.371	0.310	0.690	0.265
Condition * CCA	0.257	0.743	0.117	0.883	0.383
Condition * SA	0.257	0.743	0.032	0.968	0.096
Condition * TA	0.257	0.743	0.063	0.937	0.194

The second and third columns show the inclusion and exclusion prior probabilities of the parameter. The fourth and fifth columns show the posterior probabilities of including and excluding the parameter after seeing the data. Lastly, the inclusion BF (BFincl) encodes the BF for each predictor across all matched models simultaneously and quantifies the change from prior inclusion odds to posterior inclusion odds. Condition: Baseline, Causes, Cosnequences; CCA, climate change anxiety; SA, state anxiety; TA, trait anxiety.

We hypothesized a sensitivity effect for which participants with higher CCA would be more affected by the environmental framings and show the highest eco-fear scores. However, we found evidence against all three interaction terms, disconfirming our hypothesis.

Because CCA is composed of a functional and a cognitive subscale, we were interested in exploring which of these two components was more involved in the onset of climate change-related fear in response to different framings. The Bayesian ANCOVA, in which we included the two subdimensions as covariates and condition as between-subject factor, reveals that the best model predicting fear scores combines Condition and CCAfun (posterior odds of 0.421, BF_M_ = 8.713 with 0.908% error). Particularly, the analysis of effects (Table 3) shows anecdotal evidence in favor of the functional impairment subdimension (posterior odds of 0.821 with BF_incl_ = 2.861), compared to the cognitive emotional one (posterior odds of 0.472 with BF_incl_ = 0.559), in explaining fear levels. In this case, anecdotal evidence indicates an increase in posterior odds, however that the evidence is only limited and care must be taken in drawing definitive conclusions. Like the previous analysis we investigated the presence of a susceptibility effect related to one dimension or the other. Therefore, we also included two other covariates modeling the interaction between the two subdimensions and the Condition predictor. Interestingly, both of these showed a strong decrease in posterior odds, thus not supporting our expectations.

**Table 3:** Analysis of effects summarizes the effect of the different predictors (condition, cognitive and functional subdimensions of CCA) have on fear levels.

Effects	P(incl)	P(excl)	P(incl|data)	P(excl|data)	BF_incl_
Condition	0.692	0.308	0.884	0.116	3.402
CCAcog	0.615	0.385	0.472	0.528	0.559
CCAfun	0.615	0.385	0.821	0.179	2.861
Condition * CCAcog	0.231	0.769	0.052	0.948	0.183
Condition * CCAfun	0.231	0.769	0.072	0.928	0.257

The second and third columns show the prior probabilities of including and excluding the parameter in the model. The fourth columns show the posterior probabilities of including and excluding the parameter after seeing the data. Lastly, the inclusion BF (BF_incl_) encodes the BF for each predictor across all matched models simultaneously and quantifies the change from prior inclusion odds to posterior inclusion odds. Condition: Baseline, Causes, Cosnequences; CCAcog, cognitive impairment subdimension; CCAfun, functional impairment subdimension.

### 3.3 Intentions to act predictors

Our second aim was to investigate whether the framings would effectively increase people’s motivation to engage in pro-environmental action by influencing people’s eco-emotions and beliefs about climate change.

**Figure 4:**
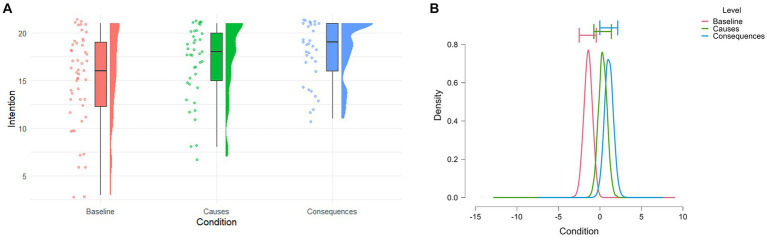
**(A)** Raincloud plot of the observed intention to act scores in the three Conditions. **(B)** Posterior distributions of the effect of each condition on fear scores. The horizontal error bars represent 95% credible intervals.

Therefore, we first conducted a Bayesian ANOVA on intentions scores among the three groups. Because of the particularly high social relevance of climate change issues and the widespread diffusion of this topic, we added social desirability scores as a random factor, thus including it in the null model. Results revealed strong evidence in favor of an effect of condition on intention scores (posterior odds of 0.871, BF_10_ = 6.78 with 0.539% error). The post-hoc comparisons revealed that the only consistent evidence in favor of a difference between groups was found for the Consequences vs. Baseline comparison (adjusted posterior odds of 4.962, BF_10,U_ = 8.447 with 4.377×10^−7^% error). Conversely, the Causes framing did not motivate action consistently more than the Baseline (adjusted posterior odds of 0.596, BF_10,U_ = 1.015 with 0.012% error) or than the Consequences framing (posterior odds of 0.266, BF_10,U_ = 0.453 with 0.013% error). We hypothesized the Causes only condition to motivate action the most, however this was not the case, and the results resembled the differences between framings found on fear scores (see Figure 4) suggesting a consistent relevance of fear in predicting intentions. Therefore, we used Bayesian Multimodel inference to investigate which factors predicted intentions to act in the three different framings. For each condition, we first conducted a correlation analysis to explore the relationship between the scores in all tests and intentions to act. Here we included all the scores considered in the previous analyses plus the Climate Change Perception scale scores.

Specifically, we selected all predictors that demonstrated evidence in favor of a linear relationship with intention-to-act scores. To do so, we conducted a linear correlation analysis, and we selected the parameters that showed at least moderate evidence (BF_10_ > 3.00) in favor of a bidirectional linear relationship with intention to act scores, independently of the strength of correlation (assessed with Pearson’s *r*). Lastly, given our main focus on CCA, we added CCA in all regressions, independently of the correlation results, as well as the Fear x CCA interaction term as possible predictors.

#### 3.3.1 Baseline

The correlation analyses demonstrated that the following factors were correlated with intention scores: CCA (*r* = 0.401; BF_10_ = 10.152), CCP-R (*r* = 0.589; BF_10_ = 3326.527), CCP-NC (*r* = 0.427; BF_10_ = 18.412), Fear (*r* = 0.583; BF_10_ = 2510.331) and Anger (*r* = 0.388; BF_10_ = 7.559). Thus, all these predictors were included in the multiple regression as covariates (See the complete analyses on OSF for diagnostics). The best model explaining intention to act scores was the one combining CCP-R and climate change related Fear scores (posteriors odds of 0.174, R^2^ = 0.473). Specifically, the model-averaged posterior summary table (Table 4) shows comparable evidence only for CCP-R and Fear as predictors of intentions (posterior odds of 0.921 with BF_incl_ = 9.588, and 0.922 with BF_incl_ = 7.070 respectively), while very weak evidence in favor of both CCA and the Fear x CCA interaction term (posteriors of 0.661, with BF_incl_ = 1.596, and 0.401 with BF_incl_ = 1.116 respectively).

**Table 4:** Model-averaged posterior summaries for multiple linear regression coefficients: Baseline.

Coefficient	P(incl)	P(excl)	P(incl|data)	P(excl|data)	BF_incl_	Mean	SD	95% Credible interval	
								Lower	Upper
Intercept	1.000	0.000	1.000	0.000	1.000	14.980	0.506	13.986	16.036
CCP-R	0.550	0.450	0.921	0.079	9.588	0.655	0.330	−0.011	1.183
CCP-NC	0.511	0.489	0.444	0.556	0.765	0.085	0.173	−0.190	0.473
Fear	0.625	0.375	0.922	0.078	7.070	0.695	0.483	−0.047	1.774
Anger	0.475	0.525	0.379	0.621	0.676	0.012	0.082	−0.155	0.247
CCA	0.550	0.450	0.661	0.339	1.596	0.262	0.332	−0.024	1.105
Fear * CCA	0.375	0.625	0.401	0.599	1.116	−0.015	0.025	−0.081	0.007

The first column lists all predictor included in the regression; P(incl) and P(excl) represent the prior inclusion and exclusion probabilities, respectively; P(incl|data) and P(excl|data) represent the posterior inclusion and exclusion probabilities. BF_incl_ is the inclusion Bayes factor which quantifies the change from prior to posterior odds, Mean and SD are the posterior mean and standard deviation of the parameter following model averaging. The last two columns represent the 95% credible interval (CI) for each parameter. CCP-R, Reality subdimensions of the climate change perception scale; CCP-NC, Negative consequences subdimension of the climate change perception scale; Fear, climate change-related fear; Anger, climate change-related anger; CCA, climate change anxiety.

#### 3.3.2 Causes

The correlation analyses demonstrated that the following factors were consistently correlated with intention scores: CCP-NC (*r* = 0.399; BF_10_ = 4.203), Fear (*r* = 0.593; BF_10_ = 405.497), Anger (*r* = 0.488; BF_10_ = 23.792). The results of the multiple regression that included these predictors revealed that the best model was the Fear only one (posterior odds of 0.246, R^2^ = 0.351). Indeed, the BMA analysis of coefficients showed consistent evidence only in favor of fear scores as a predictor of intention scores (posterior odds of 0.951, BF_incl_ = 13.896) compared to the other factors (see Table 5).

**Table 5:** Model-averaged posterior summaries for multiple linear regression coefficients: Causes.

Coefficient	P(incl)	P(excl)	P(incl|data)	P(excl|data)	BF_incl_	Mean	SD	95% Credible interval	
								Lower	Upper
Intercept	1.000	0.000	1.000	0.000	1.000	16.744	0.499	15.725	17.748
CCP-NC	0.512	0.488	0.432	0.568	0.725	0.080	0.166	−0.141	0.523
Fear	0.581	0.419	0.951	0.049	13.896	0.249	0.503	−0.872	0.983
Anger	0.512	0.488	0.514	0.486	1.010	0.077	0.114	−0.067	0.355
CCA	0.535	0.465	0.529	0.471	0.977	−0.054	0.172	−0.470	0.280
Fear * CCA	0.349	0.651	0.361	0.639	1.055	0.011	0.019	−0.008	0.058

The first column lists all predictor included in the regression; P(incl) and P(excl) represent the prior inclusion and exclusion probabilities, respectively; P(incl|data) and P(excl|data) represent the posterior inclusion and exclusion probabilities. BF_incl_ is the inclusion Bayes factor which quantifies the change from prior to posterior odds, Mean and SD are the posterior mean and standard deviation of the parameter following model averaging. The last two columns represent the 95% credible interval (CI) for each parameter. CCP-NC, Negative consequences subdimension of the climate change perception scale; Fear, climate change-related fear; Anger, climate change-related anger; CCA, climate change anxiety.

#### 3.3.3 Consequences

The correlation analyses demonstrated that the following factors were correlated with intention scores: CCP-TD (*r* = −0.546; BF_10_ = 38.496), Fear (*r* = 0.636; BF_10_ = 420.363), Anger (*r* = 0.668; BF_10_ = 1225.547). Thus, these were included in the multimodel bayesian regression as predictors of intention to act following the Consequences condition. The best model resulted to be the one CCP-TD + Anger model (posterior odds of 0.175, R^2^ = 0.572). Table 6 shows the model averaged posterior estimates for each parameter. Indeed, CCP-TD and Anger (posterior inclusion odds of 0.834, BF_incl_ = 4.566 and 0.858, BF_incl_ = 5.197 respectively) best predict intention to act scores in the Consequences condition and are the only showing positive inclusion BFs.

**Table 6:** Model-averaged posterior summaries for multiple linear regression coefficients: Consequences.

Coefficient	P(incl)	P(excl)	P(incl|data)	P(excl|data)	BF_incl_	Mean	SD	95% Credible interval	
								Lower	Upper
Intercept	1.000	0.000	1.000	0.000	1.000	17.758	0.382	17.036	18.697
CCP-NC	0.487	0.513	0.349	0.651	0.563	0.033	0.154	−0.241	0.475
CCP-TD	0.523	0.477	0.834	0.166	4.566	−0.231	0.143	−0.461	0.000
Fear	0.613	0.387	0.586	0.414	0.896	0.253	0.387	−0.124	1.270
Anger	0.538	0.462	0.858	0.142	5.197	0.207	0.129	0.000	0.422
CCA	0.548	0.452	0.501	0.499	0.828	0.082	0.200	−0.059	0.709
Fear * CCA	0.362	0.638	0.219	0.781	0.494	−0.004	0.016	−0.044	0.020

The first column lists all predictor included in the regression; P(incl) and P(excl) represent the prior inclusion and exclusion probabilities, respectively; P(incl|data) and P(excl|data) represent the posterior inclusion and exclusion probabilities. BF_incl_ is the inclusion Bayes factor which quantifies the change from prior to posterior odds, Mean and SD are the posterior mean and standard deviation of the parameter following model averaging. The last two columns represent the 95% credible interval (CI) for each parameter. Note that higher CCP-TD scores represented a higher belief that the consequences of CC would happen only further away in time. CCP-NC, Negative consequences subdimension of the climate change perception scale; CCP-TD, Temporal distance subdimension of the climate change perception scale; Fear, climate change-related fear; Anger, climate change-related anger; CCA, climate change anxiety.

## 4 Discussion

In this study, we examined the role of CCA in shaping fear toward the consequences of climate change, by analyzing how messages that present varying perspectives on the causes and consequences of climate change impact individuals. In addition, our goal was to delve into the influence of fear on individuals’ inclination to take decisive measures. This study offers a unique perspective in analyzing the distinct effects of the cognitive and functional subdimensions of CCA on the process of fear formation. Moreover, it aims to differentiate these effects from general measures of anxiety, such as state and trait anxiety.

Following a Bayesian approach, we found that our manipulation specifically increased eco-fear (and not eco-anger), confirming our hypothesis for which the Consequences condition would induce the highest fear scores. However, this was true only compared to the Baseline message, while the comparison with the Causes condition indicated that these induced the same fear levels (Figure 2), differently from what we expected based on van Zomeren et al.’s results, from which the messages used in the environmental framings were adapted.

Indeed, the mood manipulation results confirm what we expected about the Consequences condition as being the most effective in inducing decreased emotional valence and increased emotional arousal, possibly indicating that these differences in mood did not translate in increased eco-fear. However, we speculate that the fear-inducing effect of reading about the causes without specifying the consequences could be due to an anticipatory response. This proposition supports the consistent reduction of negative affect values observed between the pre-test and post-test phases, as indicated by the significant Time x Condition interaction. Notably, post-hoc analyses yielded moderate evidence of increased negative affect solely in the Causes condition, as opposed to the Consequences and Baseline conditions. Therefore, the observed negative affect increase may signify the presence of such an anticipatory effect, which may account for the disparity in results compared to van Zomeren et al. and could be associated with the heightened awareness of climate change issues over the past decade (Moser, 2016). It may also be influenced by the particular sensitivity and awareness of our predominantly young adult sample, given that young adults tend to exhibit increased sensitivity and awareness of climate-related issues (European Commission, 2009).

### 4.1 Climate change anxiety and climate change-related fear

As expected, we found that participants’ dispositional CCA levels interacted with the differently framed messages to increase CC-related fear. Indeed, Bayesian model comparison revealed that the combined effect of condition and CCA best explained the observed fear levels. Additionally, we found this effect to be specific for CCA and not for state or trait anxiety levels, for which there was no evidence of any predictive power on fear levels (see Figure 3). Indeed, the analysis revealed extreme evidence in favor of an effect of CCA on fear levels and no evidence supporting an effect of either state or trait anxiety levels (Table 2). While previous studies underline a positive relationship between GAD-related symptoms in predicting higher CCA scores (Innocenti et al., 2021; Whitmarsh et al., 2022; Asgarizadeh et al., 2023; Schwartz et al., 2023). This evidence is consistent with previous accounts that discuss CCA as being distinct from generalized anxiety-related manifestations. Notably, while we found evidence confirming our hypothesis, we did not find evidence supporting a “sensitivity” effect. Namely, we expected to observe that participants showing the highest levels of dispositional CCA would be the most susceptible to the highest fear-inducing framing (initially hypothesized to be the Consequences condition), however, this was not the case as we found evidence against all of the predictors modeling the interactions between condition and the three anxiety scales (Table 2). The strong evidence supporting CCA as directly predicting eco-fear independently of the type of framings may be due to the limited fear-inducing effectiveness of the framings, only evident in the Consequences vs. Baseline comparison. Conversely, the lack of a CCA x condition interaction can be interpreted as evidence supporting the robustness of this construct in predicting eco-fear independently of simple environmental appeals.

After confirming our first hypothesis, we were interested in exploring whether the two CCA subdimensions differently affected fear levels. Indeed, the analysis showed that the combined effect of condition and functional impairment best predicted fear levels, as confirmed by the analysis of effects showing anecdotal evidence in favor of an effect of the functional subdimension in predicting CC-related fear levels (Table 3) compared to the cognitive-emotional one. Taken together, these results seem to indicate greater importance of the functional impairment subdimension in predicting fear scores. However, the supporting evidence is only limited, in contrast with the strong evidence supporting total CCA scores as a predictor of eco-fear, possibly indicating the importance of both the subscales combined in predicting fear.

This finding is contrast with our expectations based on previous accounts. For example, Clayton and Karazsia (2020) demonstrated a selective effect of framing on the cognitive impairment compared to the functional one. Recently, Innocenti et al. (2023) referred to Wells’ model of generalized anxiety (Wells and Leahy, 1998), which states that the functional impairment follows the cognitive one and assumed that the same happens for the two subdimensions related to CCA. Even though the present study did not directly investigate the directionality effect between the two dimensions and cannot confirm nor disconfirm Innocenti and colleagues’ assumption, the results do not show a clear dissociation between the two subdimensions, indicating that both may play a consistent role in predicting eco-emotions. Similar to the previous analysis, we did not find evidence in favor of any term modeling the interaction between the two subdimensions and the condition term.

### 4.2 Intentions to act predictors

After assessing the contribution of CCA in fear formation, we were interested in measuring whether this translates into differences in intentions to act toward the environment. Interestingly, since we initially expected the Causes only framing to induce moderate levels of eco-fear, we expected this condition to be the most effective in motivating action in the absence of any efficacy framing. However, we found the Consequences framing to be the most action-motivating and the only showing a consistent difference with the neutral Baseline condition. Summarizing the previous results on eco-fear: reading about the causes and the consequences of climate change, thus manipulating anticipation of environmental threat, was the only type of framing having a consistent effect on eco-fear and, in turn, in motivating pro-environmental action intentions, seemingly suggesting a direct role of eco-fear in motivating pro-environmental action. The three Bayesian multiple linear regressions on each framing helped us disentangle how the messages modulated individuals’ perceptions of climate change and eco-emotions, and how these predict action intentions while also considering CCA.

Interestingly, the results show that following a neutral non-environmental framing (i.e., Baseline condition), participants’ intention to act are positively predicted both by the extent to which they belief that climate change is real and by their baseline eco-fear. On the other hand, in the Causes framing the only and strongest predictor of intentions scores is eco-fear, despite focusing on objective causes and supposedly non-emotional factors related to climate change. This finding, together with the consistent increase in negative affect scores found exclusively for the Causes framing may reflect anticipation and uncertainty surrounding the perceived consequences inferred by participants, as previously discussed.

Notably, the only consistent predictor of intention scores in both these two framings, not effective in motivating action, is eco-fear. In contrast, following the Consequences framing, which instead increased action intentions compared to the neutral framing, the best predictors were the individual’s perceived temporal proximity (CCP-TD) and eco-anger, instead of fear. Regarding the first predictor, higher CCP-TD reflected a stronger belief that the consequences of climate change would happen farther away in time, and, coherently, was found to be negatively correlated with intention scores. This finding aligns with previous research demonstrating that the closer individuals perceive climate change consequences, the stronger their intentions to take action will be (Liberman and Trope, 2008; Wang et al., 2019). On the other hand, the finding that eco-anger best predicted the data compared to fear is an unexpected result with significant implications. Specifically, the framework of the dual pathway model of coping with collective disadvantage has been proposed to explain the dynamic process of emotional coping in which both group-based anger and group efficacy are central nodes of feedback reappraisal loops, leading to collective action (Lazarus, 1991; van Zomeren et al., 2012). However, the same model has been adapted to environmental action, for which individuals cope with the collective climate crisis by regulating their fear, instead of anger, in response, to the appraised negative consequences of climate change, depending on their group efficacy beliefs. Indeed, this led to the proposal of eco-fear as a central and strong motivator of pro-environmental action (van Zomeren et al., 2010). However, our results seem to support the first model: finding that anger is the most important predictor of intentions, following the only effective framing in motivating action, supports a crucial involvement of eco-anger, even in the context of environmental collective action. This finding is coherent with the pattern of results emerging from our affective manipulation, in which we found a consistent difference in emotional arousal following the Consequences framing compared to the causes-only framing, possibly indicating the pro-active nature of eco-anger that leads to action motivation.

Previous accounts argue for a dual effect of fear on intentions: on the one hand, low levels of fear may motivate action, while high fear levels may induce eco-paralysis (van Zomeren et al., 2010; Chen, 2016). Therefore, communicating the imminent negative consequences of climate change possibly leads to ineffective defensive responses such as distancing, helplessness, and denial (Witte and Allen, 2000; Kollmuss and Agyeman, 2002; Fritsche et al., 2012; Hornsey et al., 2016; Salomon et al., 2017). However, even in conditions of high perceived fear of threat, such a pattern could be overturned if the appeals are accompanied by efficacy messages. Specifically, it has been proposed that collective efficacy appeals are more effective than individual self-efficacy messages (Chen, 2016). Specifically, Jugert et al. (2016) demonstrated that collective efficacy motivated pro-environmental intentions only through greater perceived self-efficacy, only when participants considered individual action effective in coping with large-scale environmental crises. In turn, inducing low self-efficacy levels made collective efficacy ineffective in motivating action.

On the other hand, the relationship between CCA and perceived efficacy is not straightforward. Previous evidence argues for a positive relationship between CCA and efficacy (Homburg and Stolberg, 2006; Howell et al., 2016; Helm et al., 2018; Maran and Begotti, 2021). However, Innocenti et al. (2023) recently argued for a double effect of the CCA cognitive subdomain on pro-environmental behaviors, measured with the PEB scale (Markle, 2013; Menardo et al., 2020): motivating PEBs on one side, but also inducing eco-paralysis by first negatively influencing self-efficacy levels on the other.

Another important finding emerging from these analyses is that CCA and the Fear x CCA interaction term showed only weak and limited evidence in predicting intentions scores, only following the non-environmental framing. This finding adds to the previous literature trying to disentangle the unclear relationship between CCA and behavioral engagement. Indeed, in contrast to the findings of Innocenti et al. (2021) and Stanley et al. (2021) report that eco-anxiety and eco-depression are less adaptive and relate to lower well-being compared to eco-anger, which, in turn, leads to greater involvement in pro-climate activism and personal actions. In their study, eco-depressed people were more likely to participate in climate action, while eco-anxious people were less likely to join, concluding that studying eco-anxiety in isolation from other eco-emotions would bring to the wrong conclusion that it enhances behavioral engagement. Overall, our results are in line with this proposal, given that the multiple regression results show a lack of evidence in favor of CCA as a predictor of intentions, while we observe fear and anger as being the only consistent predictors of action intentions.

Again, in the original proposal of the CCA scale, Clayton and Karazsia (2020) also found that behavioral engagement (assessed with a combination of items measuring both participants’ intentions and behaviors) was not associated with neither specific climate nor general anxiety responses. However, previous accounts have proposed that CCA, and eco-anxiety more generally, is an appropriate response to the environmental threat (Clayton and Karazsia, 2020; Clayton et al., 2023), and that could be thought of as a proactive emotion: useful in maintaining alertness on environmental challenges, soliciting cognitive engagement and increasing PEBs (Kurth and Pihkala, 2022; Schwaab et al., 2022). As such, it has been referred to as a “practical anxiety,” related to the anxiety of uncertainty about the right thing to do, possibly leading to a resolution of the problem (Pihkala, 2020; Kurth and Pihkala, 2022). In this sense, and given our and previous results, CCA may have a predictive effect on actual behaviors but not intentions to act. Not only, varying degrees of CCA may have opposite effects on intentions and behaviors. Specifically, Heeren et al. (2023) argue for the duality of the cognitive-emotional component of CCA, possibly leading to adaptive (i.e., PEBs) or maladaptive (i.e., functional impairment) responses, perhaps explaining why the functional impairment best predicted fear in our results, which, in turn, predicted action intentions only following the ineffective framings, thus not leading to an adaptive response. Based on this proposal, different dimensions of CCA may be selectively related to different eco-emotions and, therefore, the adaptive side of CCA may be put at use by understanding which types of framings and contexts tap into it to better motivate action while avoiding to pathologize eco-anxiety and incurring in increased psychological distancing (Reser et al., 2012; Clayton, 2020).

### 4.3 Limitations and future perspectives

Although the present study presents a promising outlook for eco-sustainability and proactive environmental behavior. It is crucial to recognize and address the limitations that might affect the overall conclusions. These limitations must be thoroughly examined in future research to ensure the validity and robustness of the findings. Starting with the methods, it has to be noted that the Climate Change Perceptions scale (CCPS) was originally developed in English, and the one we used here was translated by the authors and back translated by an English translator. Therefore, the scale is not validated in Italian, and care must be taken in interpreting the results. Although participants provided their city of residence, we did not explicitly ask them where they lived and had been living at the time of the experiment. People from rural or urban communities could manifest different sensitivities to climate change-related issues, although previous research did not find consistent differences in PEBs between the two (e.g., Berenguer et al., 2005; Sheasby and Smith, 2023). Therefore, this aspect should be considered in future studies. Another point that must be considered is about participants’ efficacy beliefs. As already discussed in the text, efficacy scores have been found to modulate intentions and behaviors effectively. Here, we did not assess participants dispositional efficacy about climate change, which could have shown specific patterns in both fear and intention formation. Lastly, given the limited effectiveness of textual framings in eliciting an emotional response, future studies should consider different types of framings (e.g., using videos) to investigate the relationship between CCA, e and behaviors.

## 5 Conclusion

The presented study demonstrated the specific contribution of participants’ dispositional climate change anxiety (CCA) in predicting eco-fear formation in response to differently framed environmental framings, while general trait and state anxiety measures did not. The absence of an interaction effect between framing and CCA suggests that people with higher dispositional eco-anxiety do not necessarily exhibit increased susceptibility to different messages framing threat and anticipation, possibly due to the need for more effective messaging but also indicating the robustness of this construct. Interestingly, while we observed similar patterns related to the Consequences framing in consistently increasing fear and intentions to act, compared to the Baseline, the best predictor of intentions flowing this framing was eco-anger, with important implications for the emotion-coping models of climate action and in line with previous accounts on fear, in the absence of an efficacy framing. Lastly, we highlight the unclear relationship between CCA, other eco-emotions, efficacy, and behavioral engagement, and we conclude by arguing that CCA may serve as an adaptive response directly related to actual behaviors, emphasizing the importance of not pathologizing this adaptive reaction.

## Data availability statement

The original contributions presented in the study are publicly available. This data can be found here: https://osf.io/sydz2/.

## Ethics statement

The studies involving humans were approved by Institutional Review Board, Department of Psychology, Sapienza University of Rome. The studies were conducted in accordance with the local legislation and institutional requirements. The participants provided their written informed consent to participate in this study.

## Author contributions

AvG: Conceptualization, Formal analysis, Methodology, Resources, Visualization, Writing – original draft. GF: Conceptualization, Investigation, Resources, Writing – review & editing. LP: Conceptualization, Project administration, Supervision, Writing – original draft, Writing – review & editing.
